# Associations between serum tumor necrosis factor-alpha, hippocampal-prefrontal-ventricular volumes, and clinical profiles in schizophrenia: a biomarker and neuroimaging study in North Sumatera, Indonesia

**DOI:** 10.3389/fpsyt.2026.1768855

**Published:** 2026-05-21

**Authors:** Mustafa M. Amin, Haflin Soraya Hutagalung, Dessy Mawar Zalia, Cindy Chias Arthy, Roger Ho

**Affiliations:** 1 Department of Psychiatry, Faculty of Medicine, Universitas Sumatera Utara, Medan, Indonesia; 2 Department of Neurology, Faculty of Medicine, Universitas Sumatera Utara, Medan, Indonesia; 3 School of Medicine, Hong Kong University of Science and Technology, Hong Kong, Hong Kong SAR, China; 4 Department of Psychological Medicine, Yong Loo Lin School of Medicine, National University of Singapore, Singapore, Singapore

**Keywords:** Asian population, MRI, neuroinflammation, schizophrenia, TNF-α

## Abstract

**Introduction:**

Schizophrenia is a neurodevelopmental disorder with systemic inflammation that is thought to play a role in its pathophysiology. The pro-inflammatory cytokine tumor necrosis factor-alpha (TNF-α) has been reported to be dysregulated in individuals with schizophrenia, but its relationship with brain volume changes remains inconsistent. This study aimed to analyze differences in TNF-α levels and brain volume (hippocampus, prefrontal cortex, and lateral ventricles) and their correlations in schizophrenia patients compared to healthy controls.

**Methods:**

This analytical cross-sectional investigation was conducted on 50 schizophrenia patients and 50 healthy Batak controls at the Prof. M. Ildrem Provincial Mental Hospital, Medan. Serum TNF-α levels were measured by ELISA, while brain structure volume was measured by 1.5 T MRI and analyzed using AnalyzePro software.

**Results:**

TNF-α levels in the schizophrenia group were significantly lower (3.35 pg/dl) than in the control group (16.90 pg/dl). No significant differences in hippocampal and prefrontal cortex volume were found between the groups. However, lateral ventricle volume was significantly larger in schizophrenia. Correlation analysis showed a weak negative relationship between TNF-α and prefrontal cortex volume only in the schizophrenia group.

**Discussion:**

The finding of low TNF-α in schizophrenia supports the complexity of immune dysfunction in schizophrenia, which may be influenced by medication or disease phase. Consistent ventricular enlargement reinforces previous neuroanatomical findings. The association of TNF-α with the prefrontal cortex only within the schizophrenia cohort indicates a specific interaction between inflammation and brain morphology in this patient population.

**Conclusions:**

The findings support TNF-α dysregulation in schizophrenia, albeit at lower levels. Lateral ventricular enlargement was consistently found, while changes in hippocampal and prefrontal cortex volume may be more subtle in this population. The negative correlation between TNF-α and the prefrontal cortex in schizophrenia suggests a potential role for inflammation in the pathology of this brain region.

## 1 Introduction

Schizophrenia is a mental condition that typically presents itself in the later stages of adolescence or in the early years of adulthood, with approximately 21 million sufferers worldwide (1). Although schizophrenia is treatable, many patients do not receive appropriate treatment, preventing them from functioning optimally in society (2). The precise etiology of schizophrenia remains unidentified, although several hypotheses have been suggested regarding its etiology (3).

Three main theories about the etiology of schizophrenia have been proposed, namely schizophrenia as a neurodevelopmental disorder, a neurodegenerative disorder, and a progressive neurodevelopmental disorder. Based on the available evidence, the first theory, namely schizophrenia as a neurodevelopmental disorder, is more widely accepted. This disorder is thought to begin in the first or second trimester of pregnancy and can be triggered in adolescence or young adulthood by the activation of pathological neural circuits (4). Some evidence supporting this theory includes enlarged brain ventricles in schizophrenic patients with a history of obstetric complications (5), as well as decreased brain volume in the thalamus, frontal lobe, hippocampus, gray matter, and superior posterior temporal gyrus (6).

Increased levels of TNF-α in people with schizophrenia have also been found in various studies. Several studies indicate that individuals with schizophrenia exhibit elevated levels of TNF-α compared to healthy control groups. According to a study by O’Brien and colleagues (7), schizophrenia patients had considerably higher levels of TNF-α compared to the control group. Similar findings were discovered by Czerski et al. (8), who revealed a noteworthy disparity in the levels of TNF-α between people diagnosed with schizophrenia and control subjects. TNF-α levels were also higher in schizophrenia patients with a family history of the same disorder. García-Miss et al. (9) identified greater TNF-α levels in schizophrenia compared to controls as well. Conversely, several studies have also reported lower TNF-α levels in medicated or stable-phase schizophrenia, highlighting the variability of immune profiles depending on clinical and treatment factors (10–13).

Another study by Simamora and Loebis (14) reported a substantial positive connection between TNF-α levels and disease severity, as measured using *the Positive and Negative Syndrome Scale* (PANSS). These findings indicate that TNF-α may serve as a valuable biomarker for describing the severity of schizophrenia symptoms. Research by Effendy et al. (15) also showed that TNF-α levels correlate with cognitive impairment in individuals diagnosed with schizophrenia. This study indicates that the inflammatory process reflected in TNF-α levels plays a function in the cognitive impairments often found in schizophrenia patients. More extensive studies also support the role of TNF-α in the inflammatory response associated with schizophrenia. Turhan et al. (16) found that dysregulation of TNF-α and its corresponding receptors may help explain the correlation between immune system activation and psychopathological manifestations in schizophrenia patients.

Seidman et al. (17) reported that the amygdala-hippocampus volume in patients with schizophrenia was less than that of the control group, with a decrease of 10.2%. Sim et al. (18) also found variations in hippocampus volume between individuals with schizophrenia and control subjects, with the hippocampal volume in schizophrenia being smaller than in controls.

A decrease in *prefrontal cortex* (PFC) volume has also been reported in various studies. Seidman et al. (17) documented a reduction in volume in the dorsolateral prefrontal cortex (DLPFC) in chronic schizophrenia, and Galderisi et al. (19) also noted significant differences in DLPFC size in schizophrenia in comparison to control groups. Research by Andreasen et al. (20) showed that ventricular enlargement in people with schizophrenia can be observed through a Computerized Tomography Scan (CT Scan), with a larger *Ventricle-Brain Ratio* (VBR) in schizophrenia compared to controls. This ventricular enlargement is associated with symptoms like strange behavior, decreased attention, and other affective disorders.

Previous studies, although investigating schizophrenia, have not yet linked TNF-α to brain volume in schizophrenia (21–27). Overall, various studies, including those conducted in Medan, Indonesia, show increased TNF-α levels in schizophrenia patients, which correlate with symptom severity and cognitive impairment. This study seeks to find the correlation and importance of TNF-α as a potential biomarker in schizophrenia research and how it correlates with the hippocampus, prefrontal cortex, and ventricle in the levels in schizophrenia compared to controls as well (28). This may elucidate the underlying causes of this condition and facilitate the development of more effective medicines.

## 2 Materials and methods

### 2.1 Study design and setting

This cross-sectional analytical study was conducted at the Regional Public Service Agency of the North Sumatera Provincial Mental Hospital Prof. M. Ildrem’s inpatient facilities, which have a capacity of 400 beds. The study has been approved by the Health Research Ethics Committee of the Universitas Sumatera Utara No. 1198/KEPK/USU/2025. The subjects were the Batak people. TNF-α testing was conducted at the integrated laboratory of the Faculty of Medicine Universitas Sumatera Utara, while magnetic resonance image (MRI) examinations were performed at Siloam Hospital Dhirga Surya in Medan. The study took place from May to November 2025.

### 2.2 Participants

The target population was schizophrenia patients, while the accessible population was patients in the Inpatient Unit of the North Sumatera Provincial Mental Hospital. The sample consisted of patients with schizophrenia who met the inclusion and exclusion criteria. The inclusion criteria for schizophrenia were people diagnosed with schizophrenia according to ICD-10 criteria, aged 18–40 years, of Batak ethnicity, amenable and prepared to an interview. Exclusion criteria for individuals with schizophrenia: history of prior psychiatric problems, general medical issues impacting brain anatomy, and obesity. Inclusion criteria for controls include individuals aged 18–40 years, of Batak ethnicity, who were receptive and amenable to an interview, and who did not have a family history of mental disorders. Exclusion criteria for controls: history of prior psychiatric problems, general medical issues impacting cerebral anatomy, and obesity. Sequential non-probability sampling was used to collect the research sample, which involved including all participants who fit the inclusion and exclusion criteria for the study and enrolling them until the sample size was achieved.

### 2.3 Sample size

The sample size for the correlation between TNF-α levels and hippocampal, prefrontal cortex, and ventricular volume in schizophrenia and healthy controls was calculated using the following sample size formula (29, 30):


$$n={\left[\frac{Z\alpha +Z\beta }{0,5\;\ln(\frac{1\;+\;r}{1\;-\;r})}\right]}^{2\;}+3$$

n = number of subjects.

α = Type I error set at 5% for a two-tailed hypothesis.

Zα = standard value at alpha = 1.96.

β = Type II error set at 10%.

Z_β_ = standard value at beta = 1.28.

r = minimum correlation coefficient considered significant set at 0.5 (30).


$$n={\left[\frac{1,96\;+\;1,28}{0,5\;\ln(\frac{1\;+\;0,5}{1\;-\;0,5})}\right]}^{2\;}+3$$

n=38

The minimum number of subjects is 38. According to the sample size table for categorical-numerical correlative analytics, the sample size is 38 (30). The research subjects were rounded up to 50 people. For between-group comparisons (independent t-test and Mann-Whitney U test), a sample size of 50 per group is considered adequate based on similar cross-sectional studies in this field (e.g., Lv et al. (10), Zhu et al. (13)), and exceeds the minimum required for correlation analysis.

### 2.4 Ethical considerations

All participants were ≥18 years of age. Written informed consent was obtained from each participant. For participants who were married or living with family, family members were also informed and provided written consent to support the study process.

### 2.5 Clinical and laboratory assessments

Schizophrenia subjects were assessed using the PANSS interview to ensure their cooperation in participating in the study. Blood samples for TNF-α level testing were collected between 7 and 9 a.m. after the subjects had fasted. Levels of TNF-α were quantified using ELISA with the corresponding kit (31). Serum was separated by centrifugation and stored at -20 °C. The ELISA procedure involved adding reagents and samples, incubating, washing, and adding conjugate and substrate solution. After incubation, adding the stop solution caused the color to change from blue to yellow. Optical density was measured at 450 nm and corrected to 540 nm using a Thermo Fisher Scientific machine (14).

### 2.6 MRI acquisition and analysis

MRI scans were performed to assess the brain structure of subjects and healthy controls, with results interpreted by a radiologist. Brain structures were recorded in millimeters, utilizing a 1.5 T MRI apparatus with T1-weighted coronal, sagittal, and axial sections and 3D MPRAGE sequences. Images were processed using AnalyzePro software to measure the volume of the hippocampus, prefrontal cortex, and ventricles. Segmentation was performed by manual tracing after the images were aligned in 3D according to the AC-PC axis (32, 33). The boundaries of the hippocampus were defined using the ADNI method, the prefrontal cortex using a perpendicular line on the sagittal slice at the genu of the corpus callosum (32), and ventricular volume was calculated from sagittal, axial, and coronal slices (34). Segmentation results were measured in mm³ and averaged after the examination was completed (35, 36).

Manual segmentation was performed by a single rater who was blinded to the participant’s diagnosis (schizophrenia vs. control). Intra-rater reliability was assessed by re-segmenting 10 randomly selected scans after a 2-week interval; the intraclass correlation coefficient (ICC) was 0.94 for hippocampus, 0.91 for prefrontal cortex, and 0.96 for lateral ventricles, indicating excellent reliability.

### 2.7 Statistical analysis

Prior to analysis, assumptions for parametric tests were assessed. Normality was evaluated using the Shapiro-Wilk test. Homogeneity of variance was assessed using Levene’s test. Based on these assessments, independent t-tests were used for normally distributed variables (hippocampus and prefrontal cortex volumes), while Mann-Whitney U tests were applied for non-normally distributed variables (TNF-α levels and ventricular volumes). For correlation analyses, Spearman’s rank correlation was used because TNF-α levels were non-normally distributed. Effect sizes were calculated: for parametric tests, Cohen’s d was computed; for non-parametric tests, effect size r was calculated as $r=\frac{\text{Z}}{\sqrt{n}}$, with 95% confidence intervals estimated using bootstrap methods (1,000 replicates). For correlation analyses, the correlation coefficient (r) itself serves as the effect size measure. Additionally, 95% confidence intervals (CIs) were calculated for the primary outcomes to estimate precision. Variables with p < 0.25 in bivariate analyses were considered for inclusion in multivariate linear regression, following established methodological recommendations for model building (37). This conservative threshold helps avoid excluding potentially important variables that may contribute to the model in combination with other factors, particularly given the exploratory nature of this study. Prior to linear regression, the assumptions of linearity, independence of errors, homoscedasticity, and normality of residuals were assessed using residual plots and the Shapiro-- Wilk test. No serious violations were detected.

Although all patients were receiving risperidone, medication dose (mean: 3.26 mg/day) and treatment duration were not included as covariates in the primary correlation analyses, as the study was designed to investigate overall associations in a naturalistic treatment setting. This decision should be considered when interpreting the findings.

## 3 Results

Based on **Table 1**, the schizophrenia group had the most subjects, namely 38 males (76.0%), with a mean age of 35.42 (± 2.78 years), PANSS score of 96.00 (81.00-110.00), BMI of 21.57 ± 2.02, and duration of illness of 18.36 months. Meanwhile, in the control group, male subjects were also the most numerous, numbering 28 people (56.0%), with a lower age but higher BMI compared to the schizophrenia group, namely 29.38 ± 5.75 years and 23.79 ± 2.98. No inferential tests were performed on baseline characteristics.

**Table 1:** Baseline characteristics.

Variable	Group	
	Schizophrenia (n=50)	Control (n=50)
Gender		
Male	38 (76.0%)	28 (56.0%)
Female	12 (24.0%)	22 (44.0%)
Age (years)	35.42 ± 2.78	29.38 ± 5.75
PANSS^a^	96.00 (81.00–110.00)	–
Body Mass Index	21.57 ± 2.02	23.79 ± 2.98
Duration of Illness (months)^b^	18.36 ± 4.15	–
Antipsychotic type	Risperidone	–
Antispychotic dose (mg/day)^b^	3.26 ± 0.61	–

^a^Data only for the schizophrenia group; presented as median (min-max).

^b^Data only for schizophrenia group; presented as mean ± SD.

**Table 2** shows that the control group had a higher average TNF-(e) level of 16.90 (5.18-56.10) pg/dl. The Mann-Whitney U test results for the TNF-α variable showed a p-value < 0.001 (Z = –6.23, r = 0.62, 95% CI for difference in medians: 9.25 to 16.80); a significant difference in TNF-α levels exists between the schizophrenia and control groups.

**Table 2:** Comparison of TNF-α levels in schizophrenia and control.

Variable	Group		
	Schizophrenia (n=50)	Control (n=50)	P-value
TNF-α (pg/dl)	3.35 (0.65–43.80)	16.90 (5.18–56.10)	<0.001

Data presented as median (min-max). Mann-Whitney U test.

The results in **Table 3** show that the volume of the hippocampus, prefrontal cortex, and ventricles was greater in the schizophrenia group compared to the control group: 4,108.25 ± 735.83 mm³, 120,014.88 ± 20,407.00 mm³, and 15,112.96 (4,262.45–37,891.53) mm³, respectively. The unpaired t-test for hippocampus showed a p-value of 0.404 (t = 0.84, df = 98, Cohen’s d = 0.17, 95% CI for difference: –165.8 to 405.6), demonstrating no significant difference between groups. For the prefrontal cortex, the unpaired t-test gave p = 0.146 (t = 1.46, df = 98, Cohen’s d = 0.29, 95% CI for difference: –2,041 to 13,355). Ventricular volume differed significantly (Mann-Whitney U = 924.0, Z = –3.09, r = 0.31, 95% CI for difference in medians: 1,156 to 5,835; p = 0.002).

**Table 3:** Differences in hippocampus, prefrontal cortex, and ventricle volume between schizophrenia and control.

Variable	Group		
	Schizophrenia (n=50)	Control (n=50)	P-value
Hippocampus (mm^3^)	4,108.25 ± 735.83	3,988.40 ± 694.84	0.404^a^
Prefrontal cortex (mm^3^)	120,014.88 ± 20.407.00	114,357.98 ± 18,087.33	0.146^a^
Ventricle (mm^3^)	15,112.96 (4,262.45–37,891.53)	12,213.93 (4,730.21–46,492.36)	0.002^b^

^a^Unpaired t-test.

^b^Mann-Whitney U test.

Data presented as mean ± SD or median (min-max).

**Table 4** presents Spearman’s correlation coefficients between TNF-α levels and brain volumes. In the schizophrenia group, there was no significant correlation with hippocampus (r = –0.015, 95% CI –0.293 to 0.265, p = 0.920) or with ventricles (r = –0.026, 95% CI –0.303 to 0.254, p = 0.860). A weak negative correlation was observed between TNF-α and prefrontal cortex volume (r = –0.299, 95% CI –0.535 to –0.023, p = 0.035). In the control group, no significant correlations were found.

**Table 4:** Correlation analysis of TNF-α levels in hippocampus, prefrontal cortex, and ventricles.

Correlation	Group	
	Schizophrenia (n=50)	Control (n=50)
TNF-α with hippocampus	r = -0.015 (-0.293 to 0.265)	r = -0.181 (-0.442 to 0.102)
	p = 0.920	p = 0.209
TNF-α with prefrontal cortex	r = -0.299 (-0.535 to -0.023)	r = 0.092 (-0.181 to 0.362)
	p = 0.035	p = 0.527
TNF-α with ventricles	r = -0.026 (-0.303 to 0.254)	r = -0.007 (-0.285 to 0.272)
	p = 0.860	p = 0.959

Spearman’s rank correlation; values in parentheses are 95% confidence interval.

The multivariate analysis, linear regression, was performed to examine whether TNF-α levels could predict hippocampal volume in the control group (the only bivariate association with p < 0.25). The model yielded an unstandardized coefficient of –15.43 (SE = 8.01, β = –0.27, t = –1.93, p = 0.060), indicating that TNF-α levels did not significantly predict hippocampal volume in this group.

## 4 Discussion

The results of this study, which found that the number of male schizophrenia subjects was higher, namely 38 subjects (76.0%), align with the findings of the TNF-α -308 G/A polymorphism investigation by Boin et al. (38), which found that the number of male schizophrenia subjects was higher than the number of female subjects. Comparable findings were likewise documented by Tan et al. (39, 40), Czerski et al. (8), Chen et al. (41), Lv et al. (10), Hoseth et al. (42), and Suchanek-Raif et al. (43). However, different results showing a higher number of female subjects than male subjects have also been reported by Pae et al. (44), and Kampman et al. (45). This pattern is also consistent with data from Canada, which found that male schizophrenics are hospitalized more often than females (46). Several possible reasons why male schizophrenics are hospitalized more often than female schizophrenics are that, proportionally, there are more male schizophrenics than female schizophrenics, less adherence to treatment, and more frequent suicide attempts, resulting in more frequent hospitalization (47). A literature review from Li et al. (48), also concludes that male schizophrenia patients experience higher hospitalization rates than their female counterparts. because female schizophrenia patients respond better to treatment and less than 50% require inpatient care.

The mean age of schizophrenia identified in this investigation was 35.42 ± 2.78 years, which is consistent with research conducted by Häfner (49), which found that the cumulative incidence of schizophrenia was between 12 and 59 years. The US diagnostic criteria also state that schizophrenia symptoms occur in youth and the mid-30s (50).

The total PANSS score obtained in this study for the schizophrenia group was 96.00 (81.00–110.00). These results indicate that most research subjects were in the moderate-to-severe symptom category, with some individuals showing very severe symptoms (as indicated by scores up to 110). This score is consistent with the characteristics of the study population, which was taken from a psychiatric hospital inpatient facility, where patients requiring inpatient care generally experience more acute, severe, or unstable symptoms.

These high PANSS scores are consistent with several studies in hospitalized populations. A study by Leucht et al. (51) in an analysis of antipsychotic clinical trials reported that the mean baseline PANSS scores for acute inpatients often ranged from 85 to 105, reflecting a significant level of symptom severity. Furthermore, research by Owen et al. (3) established that schizophrenia necessitating hospitalization is typically linked to a significant burden of positive symptoms (including hallucinations and delusions) and negative symptoms (such as avolition and diminished emotional expression), contributing to elevated total PANSS scores. This high score reflects the severity of psychotic symptoms and indicates a high level of distress and functional impairment in patients, which is the primary clinical reason for hospitalization. Therefore, the PANSS score of 96.00 in this study provides a representative picture of the clinical severity of the schizophrenia population studied and reinforces the context that the findings regarding TNF-α and brain volume were obtained in a group of patients experiencing quite severe symptoms.

The Body Mass Index in the schizophrenia group remained within the standard range, namely 21.57 ± 2.02. As noted in the literature review by Koola (52), one factor that can affect cytokine levels in the body is obesity. Thus, the study found that the schizophrenia group with a normal BMI had a reduced likelihood of obesity-related cytokine alterations; however, this was not formally tested.

The mean duration of schizophrenia in this study population was 18.26 months (approximately 1.5 years). These findings indicate that most of the study subjects were in the early-course schizophrenia phase, which is a critical period in the course of the disease where pathophysiological processes are still active, and responses to treatment can vary greatly. This relatively short duration of illness is an important contextual factor for interpreting the study’s main findings, including the inflammatory profile (low TNF-α) and brain morphological changes (53). In the early phase, neuroinflammatory and neurodegenerative processes may not yet have reached the stable state often seen in chronic diseases.

In this study, all subjects with schizophrenia received pharmacological therapy in accordance with the national formulary standards of the Social Security Administration Agency (BPJS) Health, with risperidone as the first-line atypical antipsychotic (54, 55). Regarding therapeutic interventions, it is important to specify that, based on hospital medical records and the national formulary, all schizophrenia patients in this study were prescribed risperidone as their primary oral antipsychotic, with a mean daily dose of 3.26 mg, representing the standard of care within this public health setting. None of the participants was receiving long-acting injectable (LAI) antipsychotics or had undergone modified electroconvulsive therapy (mECT) or other intensive therapeutic interventions during the study period. However, detailed data regarding the prescription of adjunctive medications, such as trihexyphenidyl or other anticholinergic agents used to manage extrapyramidal symptoms, were not systematically collected for this analysis. Furthermore, we assessed for substance use among participants. While no concurrent use of illicit substances was reported or clinically documented, a significant majority of the male participants in both groups were habitual smokers. Detailed data on the quantity and duration of smoking, however, were not systematically collected. These represent potential confounding variables, as both anticholinergic medications and nicotine are known to have immunomodulatory effects (32, 56, 57). The use of risperidone at a dose of 3.26 mg/day in this study not only represents the standard of care in Indonesia but also becomes an important factor in the interpretation of the inflammatory and brain morphology profiles, given that antipsychotic drugs are known to modulate the cytokine system and have potential neuroprotective or neurotoxic effects.

This research found that the average TNF-α level in the schizophrenia group was 3.35 (0.65-43.80) pg/dl, which was lower than the control group’s level of 16.90 (5.18-56.10) pg/dl. Comparable findings to this study, which identified reduced TNF-α levels in the schizophrenia cohort relative to the control group, were observed in research conducted by Chiang et al. (11), which found lower TNF-α in the male schizophrenia group (1.77± 0.36 pg/dl) compared to the control group (1.82± 0.57 pg/dl). Other studies that yield findings analogous to this research include Tian et al. (12), Lv et al. (10), and Zhu et al. al (13). Research indicating elevated TNF-α levels in schizophrenia has also been reported (58–63). Multiple factors are believed to affect TNF-α levels in schizophrenia patients, including antipsychotic medication, illness severity, and vitamin D variations. Cytokines may regulate dopamine, serotonin, noradrenaline, and glutamate (64). Memory, emotion, anxiety, cognition, and neural activity can be affected by reduced levels of inflammatory cytokines in the brain (42, 65). TNF-α plays a critical role in regulating complex processes such as immunity and inflammation.

This study’s observation of reduced TNF-α levels in schizophrenic patients, in contrast to several studies indicating elevated TNF-α, may be linked to the administration of antipsychotic treatment, specifically risperidone at an average dosage of 3.26 mg/day. The findings align with studies indicating that atypical antipsychotics, such as risperidone, exert a modulatory influence on the immune response, notably by suppressing pro-inflammatory cytokines such as TNF-α. Miller et al. (32, 66) concluded in their meta-analysis that schizophrenia patients treated with antipsychotics, especially atypical ones, tend to show lower levels of inflammatory cytokines compared to untreated patients or those in the acute phase prior to treatment. The risperidone dose used in this study was within the therapeutic range that has been proven clinically effective and may contribute to the negative regulation of TNF-α production. Similar findings were reported in the study by Gürcan et al. (67). Furthermore, variations in TNF-α levels across studies may be influenced by disease phase, treatment duration, and type of antipsychotic used (57). Low levels of TNF-α can also affect synaptic plasticity (68). It remains uncertain if TNF-α present in individuals with schizophrenia serves as a marker of primary pathophysiological processes or as a secondary condition resulting from the illness mechanism (42). Low TNF-α levels in schizophrenic individuals may indicate a breakdown in the inflammatory system or deliberate suppression of these cytokines (10).

This study found that the hippocampal volume in the schizophrenia group was 4,108.25 ± 735.83 mm³ and in the control group was 3,988.40 ± 694.84 mm^³^. Comparable findings were reported by Swayze et al. (69), who found no disparity in hippocampal volume between the schizophrenia and control cohorts. A meta-analysis study showed varying outcomes (70–73). The hippocampal volume found in the schizophrenia group in this study was smaller than normative values reported in other populations (74, 75). No statistically significant differences were observed in hippocampal or prefrontal cortex volumes between groups, suggesting that volumetric changes in these regions may be subtle or less prominent in this specific population.

Furthermore, the outcomes of the unpaired t-test for the prefrontal cortex variable indicated a p-value of 0.146 (p>0.05), indicating that there was no disparity in the prefrontal cortex between the schizophrenia and the control. This study found that schizophrenia prefrontal cortex volume was 120,014.88 ± 20,407.00 mm^³^and in the control group was 114,357.98 ± 18,087.33 mm^³^. This study is supported by other studies with similar results by Zhang et al. (76). Different results were found in studies conducted by Hirayasu (77), Wible et al. (78), Breier et al. (79), and Powell et al. (80). Zhang et al. (76) estimated that the reduction in the prefrontal cortex among the schizophrenia cohort resulted from extensive antipsychotic usage and was attributable to neuroinflammatory mechanisms. A decrease in prefrontal brain volume may potentially be linked to negative schizophrenia symptoms, however this is currently debated (77).

Meanwhile, the outcomes of the Mann-Whitney U test for ventricular variables indicated a p-value of 0.002, demonstrating a notable disparity in ventricular size between the schizophrenia and the control. The results of this study found that the lateral ventricular volume was larger in the schizophrenia group, namely 15,112.96 (4,262.45-37,891.53) mm³ compared to the control group, namely 12,213.93 (4,730.21-46,492.36) mm^3^ These results are supported by previous studies that found similar results (20, 81–85). Ventricular enlargement in the schizophrenia group is a consistent and strong result reported in studies related to schizophrenia (82), estimated to be about 3 to 4 times larger than normal ageing (83), and this is thought to be associated with negative symptoms and functional impairment (20), as well as difficulty in naming objects (82). The cause of ventricular enlargement in schizophrenia is currently unknown, and ventricular enlargement is not specific to schizophrenia but can also be found in other medical or neurological conditions (82).

No significant correlation was seen between TNF-α levels and the hippocampus in schizophrenia (p=0.920), nor in the control group (p=0.209). Previous research by Chang et al. (86) demonstrated that the hippocampus is susceptible to TNF-α. The presence of increased cytokine levels in the hippocampus will trigger inflammatory damage, leading to behavioral modifications (87). Altered TNF-α levels in the brain will influence neurotrophic factor protein levels, resulting in morphological changes in the hippocampus (88) and reducing neurite growth and hippocampal branching (89).

Spearman’s statistical test in the schizophrenia study found a statistically significant connection between TNF-α levels and the prefrontal cortex (p=0.035). Higher TNF-α levels are associated with decreased prefrontal brain volumes, indicating a weak negative association. In the control group, no statistically significant association was observed (p=0.527). Prior research has determined that symptoms including depression and suicide attempts may be triggered by TNF-α in the prefrontal cortex (90). The presence of cytokines in the prefrontal cortex can also cause a decrease in language fluency in schizophrenia (91) and affect reading and attention abilities (92).

In schizophrenia, there was no significant correlation between TNF-α levels and ventricular size (p=0.860). In the control group, no statistically significant link existed (p=0.959). High TNF-α levels are thought to be neurotoxic to monoamine neurones. Furthermore, injection of TNF-α can stimulate the hypothalamic-pituitary-adrenal axis and modify the levels of the neurotransmitters dopamine, norepinephrine, and serotonin. All of these factors can influence cerebral growth and morphology (93). In general, the function of TNF-α in the central nervous system remains ambiguous. Some consider it to be neuroprotective in the morphology of the schizophrenic brain, but others claim that it has a neurotoxic effect on monoamine neurones.

The results of the linear regression analysis yielded a p-value of 0.060 (p>0.05), so statistically, TNF-α levels cannot predict hippocampal variables. This result is also consistent with the statement that TNF-α can have neuroprotective or neurotoxic effects. With its non-specific mechanism of action, it may be inferred that multiple variables influence the hippocampus, including genetics, environmental conditions, and the interplay between genes and the environment.

This study has several important methodological limitations that must be acknowledged when interpreting the findings. First, its cross-sectional methodology precludes any determination of causal relationships between TNF-α, cerebral alterations, and the initiation or progression of schizophrenia. Second, the potential for confounding factors such as disease duration, lifestyle factors, and medical comorbidities were not strictly controlled, which could affect both TNF-α levels and brain volume.

Third, a key limitation is the lack of granular data on the complete pharmacotherapy regimen. Although all patients were prescribed risperidone as their primary antipsychotic—a finding consistent with meta-analyses demonstrating that atypical antipsychotics like risperidone exert modulatory effects on pro-inflammatory cytokines (32, 57)—we did not systematically collect data on the use of adjunctive medications, particularly anticholinergic drugs like trihexyphenidyl, which are frequently co-administered to manage extrapyramidal symptoms. This is a significant consideration, as these agents may possess independent anti-inflammatory properties that could influence serum TNF-α levels and potentially confound the observed correlations with brain structure.

Fourth, we can confirm that none of the participants received long-acting injectable antipsychotics or modified electroconvulsive therapy, which helps maintain a degree of homogeneity in the treatment approach. However, the high prevalence of smoking among our participants, particularly the male subjects, introduces another potential confound. Nicotine is a well-established immunomodulator, and host factors, including smoking behavior, can significantly alter cytokine profiles (56). The absence of detailed smoking history (e.g., pack-years, current vs. past use) prevents us from controlling for its effect in our analysis. This is particularly relevant given that our schizophrenia group was predominantly male (76.0%), a demographic known to have higher smoking rates.

Fifth, the limited and homogeneous sample size of 50 participants per group, along with the exclusive focus on a single ethnicity (Batak), constrains the generalizability of the findings to a wider and more heterogeneous schizophrenia population. While this ethnic homogeneity provides valuable preliminary data for the genetic and demographic context in Indonesia, it limits broader application.

Sixth, although manual segmentation was performed by a blinded rater with good intra-rater reliability, inter-rater reliability was not evaluated. Additionally, volumetric analyses were not corrected for intracranial volume (ICV), which is common in neuroimaging studies to account for head size differences. Since ICV data were not collected, differences in absolute volumes between groups should be interpreted with caution.

In essence, while our finding of low TNF-α in this medicated, early-course schizophrenia sample is intriguing, we cannot rule out the confounding effects of adjunctive anticholinergic medications or chronic smoking. These factors, along with the primary antipsychotic therapy, may collectively contribute to the immune profile we observed. This complexity underscores the challenges of isolating disease-specific biomarkers in a clinical treatment setting and may help explain the variability in cytokine findings across studies (59).

Therefore, future investigations should aim for a more rigorous and comprehensive approach. Longitudinal studies are crucial for tracking changes in TNF-α and brain structure over time during treatment. Prospective studies are needed that meticulously document not only the primary antipsychotic but also all adjunctive medications (including type, dose, and duration). Furthermore, detailed substance use histories, particularly for tobacco, should be collected and treated as critical covariates in the statistical analysis. Future studies should also more rigorously control for and analyze the effects of medication types, illness duration, nutritional status (such as vitamin D), and other inflammatory factors. There is a need to expand biomarker and genetic assessments to investigate a broader panel of cytokines (e.g., IL-6, IL-1β) and to consider TNF-α-related gene polymorphisms to understand individual variation (23, 68). A more heterogeneous population is essential to reproduce findings in larger, multi-ethnic samples that include multiple disease stages (first-episode versus chronic) for more generalizable outcomes. Finally, functional correlations integrating neuroimaging (e.g., functional magnetic resonance imaging [fMRI]) and detailed cognitive assessments are needed to understand the clinical implications of these biomarker and structural changes for patient symptoms and cognitive function.

## 5 Conclusions

Compared to healthy controls, schizophrenia patients show significant dysregulation of the TNF-α pathway. Serum TNF-α levels fall significantly in schizophrenia, contradicting previous findings. These data substantiate the idea that immunological dysfunction in schizophrenia is intricate and does not consistently present as heightened global inflammation, potentially influenced by factors such as prolonged antipsychotic treatment, disease stage, or particular demographic traits.

The most consistent finding was an enlargement of lateral ventricle volume in schizophrenia. This reinforces strong evidence in the literature that ventricular enlargement is a robust neuroanatomical *marker* in schizophrenia. No significant volumetric differences were seen in the hippocampus and prefrontal cortex between individuals with schizophrenia and controls within the Batak population. This indicates that volume alterations in certain areas may be more nuanced, vary across populations, or be influenced by specific genetic and environmental factors.

A weak and specific negative correlation existed between elevated TNF-α levels and reduced prefrontal brain volume, exclusive to schizophrenia. This indicates that inflammatory processes involving TNF-α may exert a more significant local effect on prefrontal regions in patient groups, despite lower overall levels. This relationship was not found in healthy controls.

This exploratory cross-sectional study provides preliminary evidence of TNF-α dysregulation and lateral ventricular enlargement in schizophrenia among the Batak population. It highlights the complexity and variability of the TNF-α inflammatory profile, which is not always elevated, underscoring the importance of considering disease phase, treatment, and ethnic background. It provides valuable preliminary data from the Batak ethnic group in Indonesia, which has been underrepresented in the global psychiatric neuroscience literature. These findings also emphasize the importance of considering population-specific genetic and environmental variations when mapping schizophrenia biomarkers, and confirm that TNF-α has potential as a relevant biomarker, particularly in relation to the prefrontal area, although the mechanisms and direction of its relationship (neuroprotective vs neurotoxic) still need to be clarified. The findings should be interpreted with caution given the methodological limitations, and future longitudinal studies with larger, more diverse samples are needed to validate these observations.

## Data Availability

The datasets presented in this article are not readily available because they contain sensitive patient information, including clinical and neuroimaging data, that could compromise participant privacy and consent. De-identified data supporting the findings of this study can be made available upon reasonable request. Requests should be directed to the corresponding author, Mustafa M. Amin, at mustafa.mahmud@usu.ac.id.
